# It does not have to be either or! Assessing competence in medicine should be a continuum between an analytic and a holistic approach

**DOI:** 10.1007/s10459-021-10043-0

**Published:** 2021-03-29

**Authors:** Thomas Rotthoff, Martina Kadmon, Sigrid Harendza

**Affiliations:** 1 Medical Didactics and Educational Research, DEMEDA, Medical Faculty, University of Augsburg, Universitätsstrasse 2, 86159 Augsburg, Germany; 2 Medical Education Sciences, DEMEDA, Medical Faculty, University of Augsburg, Augsburg, Germany; 3 III. Department of Medicine, University Hospital Hamburg-Eppendorf, Hamburg, Germany

**Keywords:** Assessment, Educational, Competence, Performance, Education, Competency-based

## Abstract

Assessing competence is a tremendous challenge in medical education. There are two contrasting approaches in competence assessment: an *analytic* approach that aims to precisely measure observable constituents and facets of competence and a holistic approach that focuses on a comprehensive assessment of competences in complex real situations reflecting actual performance. We would like to contribute to the existing discourse about medical competence and its assessment by proposing an approach that can provide orientation for the development of competence-based assessment concepts in undergraduate and postgraduate medical education. The approach follows Kane's framework of an “argument-based approach” to validity and is based on insights into task complexity, testing and learning theories as well as the importance of the learning environment. It describes a continuum from analytic to holistic approaches to assess the constituents and facets of competence to performance. We conclude that the complexity of a task should determine the selection of the assessment and suggest to use this approach to reorganize and adapt competence assessment.

## Introduction

**Fig. 1:**
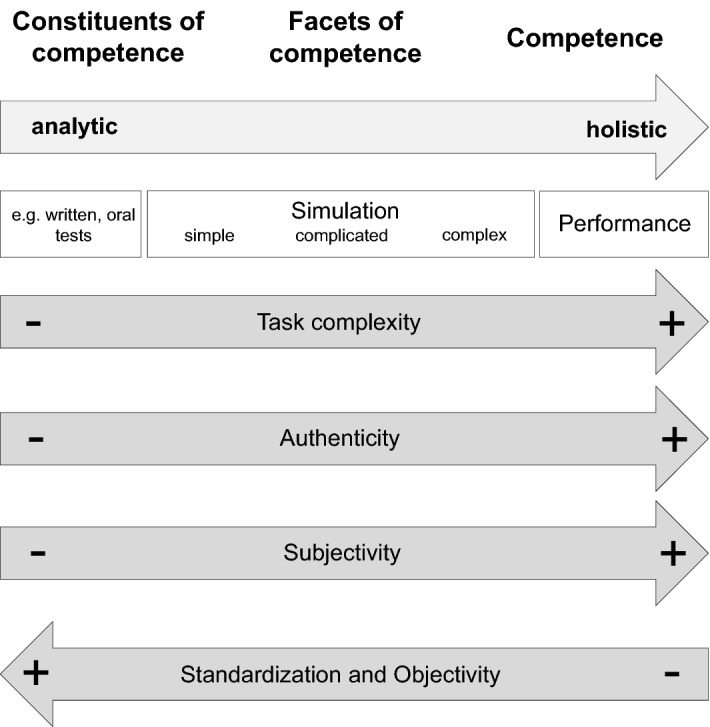
Competence Assessment Continuum Approach. Competence development results from the gradual acquisition of constituents of competence and facets of competence to competence. The assessment of competence should, therefore, be seen as a continuum from an analytic to a holistic assessment approach. Constituents of competence can easily be operationalized which provides an analytic assessment approach indirectly measuring latent competence variables by tests (e.g., Multiple-Choice Questions). Facets of competence can be tested by situational representation in simulated settings of varying complexity. Competence can be tested by observational assessment of performance in the real world. With these assessment steps, validity successively increases with respect to the actual competence of a person. Complex tasks within changing contexts result in lower operationalization, standardization and objectivity, while the relevance of the assessor’s subjectivity increases. Such settings require a more holistic approach to competence assessment. Complexity of a task should determine the selection of the assessment approach. We suggest to use this approach to reorganize and adapt competence assessment

Ultimately, the purpose of health professions education is to ensure and enhance the quality of health systems by transforming learners into qualified professionals (Govaerts, van der Vleuten, & Holmboe, 2019). Professional competence represents the basis for performing tasks of high complexity (ten Cate, Snell, & Carraccio, 2010) and is more than a demonstration of isolated competencies. It is defined as the integral use of knowledge, skills, clinical reasoning, values and reflection in daily practice for the benefit of the individual and the health care needs of the community (Epstein & Hundert, 2002). Hence, in order to prepare students to show competent behavior as physicians, it will be critical to promote and monitor the continuous development of their professional competence in performance-based assessments rather than separately assessing knowledge, skills and attitudes relevant for professional activities (Rethans et al., 2002; Lomis et al., 2016). There is a vast amount of research, on how, when and even if at all to best approach the assessment of competencies. It requires different assessment systems ‘that are standardized as well as authentic, that allow for control as well as trust, and that foster cultures that enable and value learning as well as high-quality performance.’ (Govaerts, van der Vleuten, & Holmboe, 2019). Despite an ongoing dispute about these ‘two ends of the scale,' it is a general consensus among educators that valid assessments require psychometrically sound conclusions about the latent abilities and characteristics of an individual. In particular, summative assessments are expected to fulfill psychometric test quality criteria such as objectivity, reliability and validity. 'During the second half of the twentieth century, ‘subjectivity’ became a bad word. In assessment, it was associated with unreliability. In turn, unreliability was associated with unfairness.' (Eva & Hodges, 2012). Does it still hold true in the twenty-first century that objectivity has a higher priority in assessment than the subjectivity of an experienced assessor? With our reflection, we would like to carefully consider this question and its current discussion. We are aware that our work can only provide a brief synthesis of a significant history of research and existing discourse about medical competence and its assessment. It can only touch the 'tip of the iceberg' and expand our perspective on competence-based assessment. This paper wants to advocate a thoughtful approach to assessment that considers the different strengths and weaknesses of analytic and holistic approaches. We propose the perspective of a continuum extending from an analytic approach to assessment to a holistic one (Fig. 1) and recommend using a combination of both. The choice of specific assessments along the scale between analytic and holistic will vary depending on the assessment needs and learner levels of training. Our approach is based on insights into task complexity, testing and learning theories as well as the importance of the learning environment. To discuss the approach, we will take a closer look at its two poles, 'analytic approach' and 'holistic approach,' including their constructs, definitions and conditional factors along Kane’s validity framework.

## Kane’s validity framework

Kane's validity framework defines the process of test validation as a collection of evidence to support or refute assumptions in an assessment, to interpret them, and to derive decisions that strengthen or weaken the validity argument (Kane, 2006, 2013; Cook et al., 2015). The process of validation includes the four components ‘scoring,’ ‘generalization,’ ‘extrapolation’ and ‘interpretation.’ Tests are usually based on individual observations or measurements using a score for each individual measurement. For generalizability, tests have to include multiple measurements represented by a sample of items or OSCE stations that best represent the test domain (Cook et al., 2015; Kane, 2013). Ultimately, generalization scores are used to extrapolate and to make inferences about how well a candidate would likely perform on different tasks in different contexts (Kane, 2013). These extrapolation inferences extend the interpretation to new domains of performance, i.e., the practice domain, which includes behaviors of interest in the real world (Kane, 2013). Following Kane’s framework, we first look into the analytical approach where the main focus lies on the determination of objective and reliable scores to subsequently support generalizability. We then move to the holistic approach entering a more uncertain territory. We will explore aspects like extrapolation, interpretation and decision making within the context of fidelity and authenticity.

## The two poles of assessment: analytic approach and holistic approach

The analytic approach evolved from the field of educational research and aims to support the development of individual competencies (Blömeke et al., 2015). This is based on the implicit assumption that individual elements of competence may be developed and improved by external intervention (Koeppen et al., 2008). It assumes that professional roles can be deconstructed into individual elements such as defined knowledge, skills, or attitudes, which—acquired separately—eventually lead to comprehensive competence. An analytic approach aims to precisely and objectively measure the construct to be tested by using specific methods that allow accurate and reliable rating, if repeated often enough (Blömeke, Gustafsson, & Shavelson, 2015). The purpose of such assessments is to obtain quantifying and classifying statements and assumptions about the relationship between the test behavior and the characteristic measured (Seeber et al., 2010). In any case, these tests measure latent variables that cannot be directly observed, but may only be indirectly inferred via the test approach (Seeber et al., 2010).

The assumption that deconstruction of professional roles into variables, such as defined knowledge, skills or attitudes, leads to comprehensive competencies has not yet been empirically substantiated. There is a controversial debate in the literature, since global competence may be more than just the sum of successfully completed individual tasks or demonstrated knowledge and/or skills (Brightwell & Grant, 2013; Malone & Supri, 2012; Talbot, 2004; Ashworth & Saxton, 1990). Therefore, the analytic assessment approach is not regarded as an assessment of competence in the proper sense (Hawkins et al., 2015).

In contrast, the holistic approach to competence assessment has its origin in business and organizational psychology and aims to use performance tests to predict candidates’ competence for future performance. Its purpose is not so much to assess the personal prerequisites for certain competencies, but to focus on a comprehensive assessment of competence in complex real-life situations by measuring performance (Blömeke et al., 2015). Holistic refers to the assessment of performance with respect to complex outcomes of interlinked competencies using learning objectives from different domains (e.g., knowledge, skills and attitudes). In Europe, so-called assessment centers for the selection of applicants are frequently used instead of motivational job or admission interviews. Research confirms that the holistic approach has a fairly good predictive validity for later work, but shows only a weak construct validity (Bieri & Schuler 2011; Melancon & Williams, 2006; Arthur et al., 2003; Gaugler et al., 1987; Rotthoff et al., 2014). Although assessment centers are directed toward pre-defined outcomes, they fulfill the psychometric requirements for summative testing only to a limited extent (Rotthoff et al., 2014).

With regard to the two poles of assessment, we

- …put forward our hypothesis, that assessments are not necessarily either analytic or holistic, but may be regarded as a continuum.
- …will illustrate this point by providing examples of existing assessments along the continuum between analytic and holistic.
- … will discuss the pros and cons and show what trade off (e.g., reliability for authenticity) has to be accepted as one progresses from one pole to the other, as well as discuss potential strategies for maximizing the various aspects of validity.

This reflection might help program directors, examination committees and teachers to identify the type of assessment design with respect to an assessment’s aim. To take this step in the first place has a decisive influence on the way an assessment and its rating is designed.

## Assessments in the 'real world'

### Testing with lower fidelity and authenticity: scoring and generalization

**Fig. 2:**
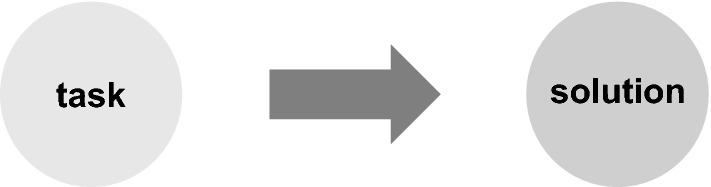
Task with a single correct solution

The analytic assessment approach aims at an accurate measurement of individual components using specific assessment methods. Fairness and objectivity, standardization and reliability are considered essential test quality criteria for such testing. Objectivity is met when performance, evaluation and interpretation are not influenced by the examiner and if independent examiners achieve the same results. This requires a high degree of standardization of the assessment and, to fulfill the criterion of objectivity, a narrow scope of correct answers and interpretation (Reetz, 2010). For a simple task with a multiple-choice Item, for example, a previously determined unique score is correct and no alternative has to be considered in most cases (Fig. 2).

**Fig. 3:**
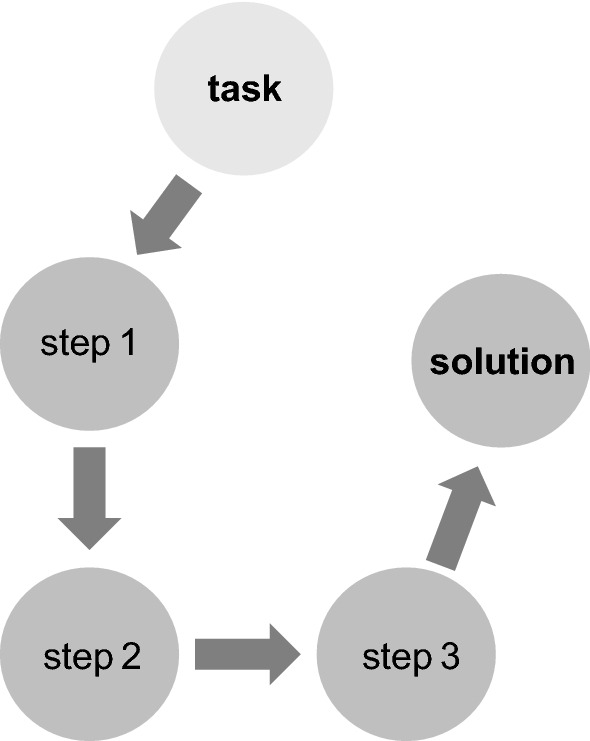
More complicated task that requires different steps to reach a single correct solution

Similarly, a single station of an Objective Structured Clinical Examination (OSCE) on a basic practical skill (e.g., taking a blood sample or providing an examination of the lungs) allows a clear operationalization of the task with an unambiguous scoring, although this is more difficult than for a multiple-choice item (Fig. 3).

Generalization tests usually consist not only of one but of multiple measurements represented by a sample of items or sample of OSCE stations representing the test domain at its best (Cook et al., 2015; Kane, 2013). We are using scores to predict future performance in some other context or to draw inferences about how well a candidate would likely perform on different tasks in different contexts (Kane, 2013). These extrapolation inferences extend the interpretation into new performance domains (Kane, 2013). However, examiners frequently complain that adding up all the scores on the individual items in a highly structured and operationalized OSCE does not necessarily show the candidates' abilities, which the OSCE is meant to assess. Operationalization of an assessment strengthens the validity argument in some aspects, but weakens it in others (Schuwirth & van der Vleuten, 2012a). The test quality criteria of reproducibility or reliability of scores can be met by using appropriate metrics. But generalization inference takes us from a sample of observations to the test-world performance and not to the real-world performance (Cook et al., 2015). We often assume, that if a candidate does well in a multiple-choice exam, he or she can apply this knowledge in practice, or if someone can demonstrate blood sampling ability on a model in a skill station, this ability can be transfered to a real person with the same quality regardless of a good or bad peripheral vein status. However, this is only an assumption, unless comparative measurement results of other observations are used for validation. It can be argued, that standardization of an assessment increases objectivity and reliability, but bears the risk of distancing the assessment from reality and authenticity, hence impeding *validity* in clinical environments (Govaerts et al., 2007). Thus, the analytic assessment approach is suitable for assessing constituents of competence, i.e., knowledge, skills and attitudes, but cannot be regarded as an assessment of competence in the proper sense (Hawkins et al., 2015). In other words, the validity of multiple individual measurements in the test-world often do not comprehensively represent the real-world construct to be measured  the final global competence of the individual (Blömeke et al., 2015). Having pointed out these limitations, let us get to a deeper insight into the holistic approach.

### Testing with higher fidelity and authenticity in simulated environments: extending interpretation to new domains of performance

Authenticity of an assessment is an important parameter for competence-based assessment to be maximally effective as an educational tool (Eva et al., 2016). Assessments in simulated environments also referred to as competence-oriented assessments—are an approximation of authentic workplace-based situations and attempt to reproduce actual professional performance demands. They seem to be an important add-on to the usual knowledge and skills tests from the more analytic approach. Simulated assessments allow the extrapolated inference that a test domain reflects the key aspects of real performance (Cook et al., 2015). Complex scenarios in simulated settings are being used when several facets of competence are assessed at the same time in order to approximate an assessment of competence (see Fig. 1), e.g., communication with a simulated patient, clinical reasoning, ordering diagnostic tests and developing a treatment plan (Prediger et al., 2020). The difficulty to operationalize the assessment of a task with respect to an unambiguous scoring grows with a task’s complexity and takes assessment further toward a holistic approach. Let us take a closer look at the meaning of complexity at this point.

The overall complexity of a task results from a component complexity, a coordinative complexity and a dynamic complexity (Wood, 1986). Component complexity is associated with the number of subtasks and information units that must be considered within a task. Component and coordinative complexity are illustrated by the following example with two assessment settings and tasks for a simulated patient with asthma.

Setting 1: *You will meet a 34-year-old patient with dry cough and sudden breathlessness.* (1) *Take a medical history of the patient regarding his current complaints. *(2) *Perform a physical examination of the lungs.* (3) *Suggest further diagnostic procedures from your findings.* In this scenario, background information on the patient’s current complaints is given in advance and every step that should be taken by the candidate during the encounter is defined separately. The candidate has to proceed through the steps in a predefined order, thus representing a component rather than a coordinative complexity.

Setting 2: *A 34-year-old patient presents for the first time in the outpatient clinic. Derive a working diagnosis on the basis of your findings and make suggestions for further diagnostics*. This scenario has more coordinative complexity because it requires more effort by the candidate to process the task including thinking about necessary actions and prioritizing them in a self-regulated process. Additionally, no indication of the patient’s symptoms is given in advance. This task, therefore, can be assigned a higher complexity due to its component and coordinative complexity, and an increasing number of steps of a task is accompanied by growing demands on the performer (Wood, 1986). With the open task formulation in setting 2, the candidate might interpret some information of the patient's medical history differently than expected, which could lead him to a different path and hypothesis resulting in alternative suggestions for further diagnostic procedures.

**Fig. 4:**
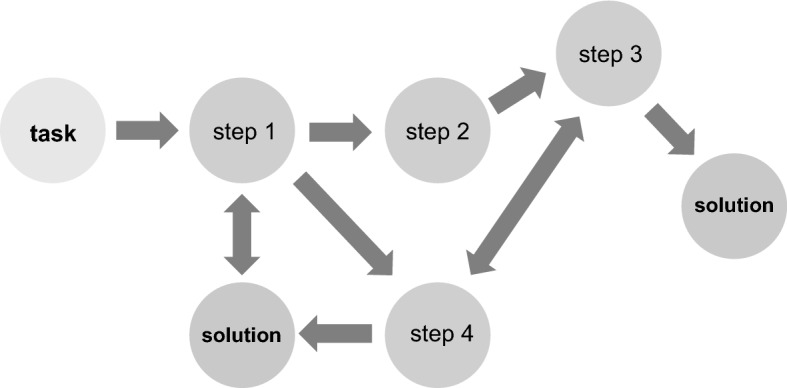
Complex task. Various paths can lead to a solution and several solutions may be correct

Dynamic complexity occurs when components are tightly coupled, governed by feedback and a time-related dimension becomes relevant (Fig. 4).

Let us take another example: A patient with an elevated cholesterol level has to decide on a therapeutic measure to lower his cholesterol. According to the guideline, both drug therapy and monitoring and reduction in other risk factors are possible. In an informed decision-making process, the physician presents the patient's relative and absolute risk for a cardiac infarction or stroke. In this situation, patients may take different decisions depending on their individual risk assessment. Their favored decision may even change during the encounter. This requires an adaptive behavior of the physician. In addition to various components and their coordination, there is also a time dimension in the decision-making process resulting from the quality of communication, information, the current risk assessment and the amount of trust established between the doctor and the patient in the course of the interaction.

Task complexity arises from the interaction between task features and the competence of the individual. Very complex tasks can become easy with sufficient practice, and pretty easy tasks can be complex for a novice. Task complexity is not merely an inherent characteristic of a task, and any assessment approach should take at least the stage of training of the individual into account. In the two settings involving the simulated patient with asthma (and especially so in the first one), the constructs to be measured are defined on the basis of a clearly and even narrowly defined role of a simulated patient with asthma. The scenario is precisely described. Therefore, assessors may hardly experience uncertainties with respect to examinees’ performance when they are in the correct state of their training for such an assessment. Standardizability of this assessment can be realized quite well for both settings by using rating scales, which provide the categories with different granularity. However, the more the rating scale tends toward a global rating, the more subjectivity of the assessor gains importance. To support the validity argument and to provide standardization, an empirical evaluation of rater accuracy and reliability by rater training is required.

### Further enhancement of fidelity and authenticity in simulated assessments

Even in simulated settings, clinical tasks and environments very close to reality can be used, e.g., a simulated first day of residency including issues on patient management, interaction with nursing staff, dealing with x-rays with false names, roster changes and handover discussion (Prediger et al., 2019, 2020; Wijnen-Meijer et al., 2013). In a competence-based assessment for flight school applicants, some competencies such as leadership, teamwork or decision-making required for complex tasks reached good comparability between different observers. This was achieved by defining specific observation anchors (Oubaid et al., 2012). Such assessment anchors can support the validity argument to get acceptable comparability of assessment results from different examiners (Prediger et al., 2020). Anchors are most influential if they are relevant to the target judgment and represent the same underlying dimension (Chapman & Johnson, 2002). An important precondition is to ensure that anchors and scales are clearly recognized, defined and understood by all assessors. Furthermore, perception errors like expectancy effects and self-fulfilling prophecies should be recognized (Krupat, 2018) and addressed in rater trainings. Although such preconditions are important they are still extremely difficult to achieve. Previous attempts to standardize complex tasks for assessment in simulated scenarios have shown ambiguous evidence for a robust prediction of performance (Goch et al., 2018; Dong et al., 2017).

Nevertheless, simulated environments can offer the possibility of representative, authentic, fair and comprehensive assessments (Wiggins, 1993), since not every learner can be offered a comprehensive set of real clinical scenarios. They may even have advantages over reality: a task may be authentic in a professional context, but not necessarily representative with respect to the specific educational goals for a given learner, thus leading to reduction in validity through an overemphasis of authenticity (Seeber et al., 2010, pp. 200–208). If, for example, a second year medical student is to take the medical history of a multimorbid elderly patient, the lack of understanding of the various clinical conditions and their relationships can certainly be overwhelming. Therefore, as mentioned above, taking the stage of training of an individual into account is an important prerequisite in competence-based assessment.

### Integrating the test‐world into the real world

Patterson et al. took it one step further and integrated a simulation into the real-world performance. They implemented resuscitation simulation scenarios into the routine working day in order to reinforce and maintain the teamwork behavior that health care personnel demonstrated in a previous laboratory-based simulated teamwork training (Patterson et al., 2013). These simulations were presented in an unannounced fashion using the inhouse paging system, and health care providers responded believing it was a ‘real’ resuscitation. Debriefing occurred immediately following the simulation. This training comes very close to reality and authenticity, and also includes *dynamic* complexity, as participants are disturbed in their current activities and have to show unprepared team work performance in a simulated resuscitation setting. Such a training is in line with the situativity theory which emphasizes that knowledge, thinking and learning are situated in a specific context or environment (Durning & Artino, 2011).

The closer we get to the real-world performance, the more limited an analytic approach to assessment becomes. To use specific and precise assessment methods which allow an accurate and reliable rating (Blömeke et al., 2015) and to make assumptions about candidate’s performance seems no longer possible. In fact, we do not want to know how well learners perform in a simulated environment, but we do want to know how they behave in real life. We will next explore this possibility.

### Assessments in the real world

Real workplace test situations are not designed with a draft, tasks may vary over time, and the final result is not fixed but may change while the task is performed (Wood, 1986). Besides this, various paths could lead to the solution, and often not just one but several solutions may be correct (Campbell, 1988) (Fig. 4). This requires ongoing adaptation of an examinee's own behavior during performance by prioritizing new information (Campbell, 1988) and integrating situation-specific different competencies for optimal patient care (ten Cate et al., 2010). It could be argued that this is also the case in simulated scenarios like in setting 2 from above with an openly formulated task ("*derive a tentative diagnosis on the basis of your findings and make a suggestion for further diagnostics*."). In this setting, the candidate also needs to prioritize new information and has to decide about the next steps. Schmidt and Mamede suggest that if the learner provides a context and content-based rationale for the next steps of their clinical decisions and actions (i.e., clinical reasoning), the examiner can better comprehend and judge an examinee’s clinical competence (Schmidt & Mamede, 2015).

### Role of the individual examiner in complex competence assessments

It has been reported that contextual changes are inseparably linked to the observer and the components of a task are considered to be a product of both the environment and the interpretation of the respective situation (Haerem, Pentland, & Miller, 2015). Individual examiners are part of a holistic validation process by assessing the learner’s performance of complexly linked tasks. The role and characteristics of the individual assessor is, therefore, considered very important (Schuwirth & van der Vleuten 2012b; Haerem et al., 2015) and subjectivity in their decision as medical experts an essential part of the assessment (ten Cate & Regehr, 2019). With the constructs to be measured becoming increasingly vague in complex real situations, examiners develop uncertainties that influence the assessment results (Scott et al., 2019). Complex tasks pose high demands on the ability of assessors to continuously adapt and evaluate changing situations and to make decisions which may limit their objectivity during the assessment. Additionally, sociology literature questions the assumption that people are rational decision makers. Instead of weighing the pros and cons of a decision objectively and logically, the model of social processes emphasizes the effects of the broader context on how decisions are made (Bruch & Feinberg, 2017). Decision-making is rather regarded as a complex iterative social process, influenced by personal experience and the views and advice of other people, and its validity is based on the degree of trust between the provider and the receiver of the information (O’Riordan et al., 2011). Therefore, context can influence an assessor’s decision about an examinee’s performance, which in turn can increase a subjective rather than an objective and standardized way of making inferences from an assessment.

### Trusting in individual expertise in the assessment situation

The concept of "Entrustable Professional Activities” (EPA) represents a paradigm between standardization and authenticity or control and trust in competence-based assessment. EPA focus on the performance of defined and interrelated complex units and of facets competence of clinical practice within the clinical environment (ten Cate & Scheele, 2007; ten Cate, 2018; Berberat et al., 2013, 2019). The decision to entrust a person with a task is always made individually by a responsible physician or by a group of clinical educators. The central question to be decided is which activity can be entrusted to a learner with which degree of independence or supervision (ten Cate et al., 2015, 2020). In everyday clinical practice, entrustment decisions are usually made without prior comprehensive structured observation of performance or objective measurements. Experience shows that a comprehensive observation of complex EPA is limited, due to their high context specificity. Decisions for entrustment are, therefore, regarded as subjective ratings by assessors (Krupat, 2018), which take additional qualities such as integrity or humility of the trainee into consideration (ten Cate et al., 2020). Empirical data on the assessment of trustworthiness of learners to act independently by clinical teachers confirm, that besides knowledge and skills discernment, conscientiousness and truthfulness of the learners were important factors (Kennedy et al., 2008). Recent studies confirm the relevance of such factors for entrustment (Prediger et al., 2020).

### Applying the 'Assessment of Competence Approach'

How can our approach support a program director or a member of an examination committee, who, for instance, wants to assess students in their final year of undergraduate training performing a ward round? What trade-off has to be accepted when progressing from the analytic to the holistic pole of our approach? How can various aspects of validity be maximized?

Due to the training level of undergraduate medical students in their final year, the assessment should take place in the real workplace and, as a first step, requires the definition of criteria for a good ward round with defined anchors. However, what is a ‘good’ ward round for the doctor may not be ‘good’ for the patient (Powell et al., 2015). The ward round could, therefore, combine patient-related and organizational tasks. To illustrate this combination of tasks, we assume that the student has to manage the discharge of a patient during the round which includes involving other health-care professionals or delegating tasks to other specialist staff. Organizational tasks within the healthcare team could comprise the coordination of appointments for possible post-hospital or outpatient check-ups, contacting and informing the institutions providing further care (e.g. rehabilitation, nursing home, nutritional counseling) and/or organizing appropriate transport according to the current condition of the patient. In direct interaction with the patient, organizational tasks could be changes in medication, arranging follow-up medication prescriptions, prescribing medical aids and providing the nursing home with instructions for further care. Such organizational tasks could be defined in advance and assessed precisely and objectively using an analytic approach by assessing steps of tasks completion or by deciding with "yes" or "no" if the task has been accomplished. Thus, with clearly defined issues to be considered in discharge management, a reliable measurement tool can be generated and provided for an analytical assessment approach. However, the quality of interaction with the team and the patient, which is required in the ‘real world’ when discharging a patient, is not adequately represented in the analytic approach. The authentic situation ideally also requires the impressions of the assessor, team members and, if applicable, the patient. Their individual perspective in assessment is relevant considering a patient's case complexity and the various issues essential for this particular discharge. For a younger patient after a successfully treated pneumonia, managing the discharge is certainly easier than for a multimorbid patient requiring nursing care.

Likewise, the assessment of communication quality with the patient will be observer-dependent including the appreciation of verbal and non-verbal signals of the patient's worries, fears and other emotions. Patients often find it difficult to understand information provided by healthcare professionals. Therefore, the intelligibility of information transfer and the interaction with the patient and healthcare team must be assessed as well, thus reflecting the holistic assessment approach. The described scenario inevitably involves an undefined assessment situation, which will be context-specific and cannot be fully anticipated in advance. All aspects can be scored by global rating, since the examiner’s subjectivity and individual experience and expertise is bound to anyway influence the assessment results.

Taken together, this assessment would include elements of both, the analytic and the holistic approach. It does not necessarily have to be either analytic or holistic, but could be viewed as being anywhere along that continuum. It requires a combination of both approaches, where the proportion of the one and the other will vary depending on the assessment goal, context and learner level of training. For the very reason that it is difficult to define and validate clear assessment criteria for individual competence and performance, we argue that we do not always have to meet the requirements of an objective assessment situation. In order to extrapolate and approximate the assessment of performance for a relevant and meaningful decision, it may often be preferred to perform several assessments with different more subjectively evaluating observers.

## Conclusion

The proposed assessment approach of competence describes the assessment of constituents and facets of competence as well as competence in the context of performance as a continuum from an analytic to a holistic assessment approach. Both approaches have their roles in assessing different aspects of competence, and they have different underlying conditions with respect to the design and types of measurements. Following Kane’s validity argument, we propose using assessments along the analytic, holistic or combined approaches depending on the assessment goal, context and learner level of training. While task complexity, authenticity and subjectivity of rating increase from an analytic to a holistic assessment approach, standardization and objectivity decrease. The analytic approach pursues objective and reliable measurements of constituents of competence with a variety of specific, mostly quantitative methods, e.g., MCQ tests, standardized structured oral assessments and clearly defined and uniquely measurable criteria. Assessments of facets of competence, which can be tested by simulation ranging from simple via complicated to complex tasks approaching holistic settings, require less standardization and objectivity, while they reach higher authenticity and validity. Additionally, more rater subjectivity may be expected, but assessment anchors can be used in order to reduce this effect. Competence of a physician can be assessed in a holistic approach by performance in the workplace. In such assessments, assessors decide subjectively whether they would entrust a task to a candidate and at what level of supervision. A higher number of expert raters can compensate the lack of standardization and objectivity in such assessment situations. No universally objective, reliable and valid test of competence exists, because competence is a context-specific construct.

Competence-based assessments can predict the prerequisites for later performance but not performance itself. Matching assessments have been designed for different aspects of competence along an academic program, and they all have their place in the continuum between an analytic and a holistic assessment culminating in the observation of performance. Competence-based assessment is not either standardization or authenticity and not either control or trust. Both approaches do not oppose each other as poles, but should be considered as intertwined.
